# Thromboembolism and bleeding risk scores and predictors of cardiac death in a population with atrial fibrillation

**DOI:** 10.5935/abc.20170064

**Published:** 2017-07

**Authors:** Rose Mary Ferreira Lisboa da Silva, Pollyana Ardavicius e Silva, Marcos Correia Lima, Lívia Tanure Sant'Anna, Túlio Corrêa Silva, Pedro Henrique Vilela Moreira, Robert Moreira Gandra, Túlio Ramos Cavalcanti, Plínio Henrique Vaz Mourão

**Affiliations:** Universidade Federal de Minas Gerais, MG - Brazil

**Keywords:** Thromboembolism/complications, Hemorrhage, Cardiovascular Diseases/mortality, Atrial Fibrillation/complications

## Abstract

**Background:**

Atrial fibrillation (AF) is a common arrhythmia, with risk of systemic embolism and death. It presents rheumatic etiology in up to 32% of developing countries, whose anticoagulation and evolution data are scarce.

**Objectives:**

to determine the predictors of cardiac death considering the clinical profile, thromboembolism and bleeding scores of patients with AF of a single center, with high prevalence of rheumatic heart disease.

**Methods:**

302 patients with AF were studied, mean age 58.1 years; 161 women; 96 pts with rheumatic etiology. Patients underwent clinical and laboratory evaluation, measurement of risk scores and the mean follow-up of 12.8 months.

**Results:**

174 were using warfarin. The averages of the HAS-BLED and ATRIA scores were 1.4 and 1.2, respectively. Percent time in therapeutic range of international normalized ratio was 45.8%. Thirty patients (9.9%) had cardiac death and 41 had some type of bleeding due to warfarin. By univariate analysis, there was statistical significance between cardiac death and permanent AF, blood pressure, systolic dysfunction, R_2_CHADS_2_, CCS, EHRA and HAS-BLED. There was no association with valvular AF. By multivariate analysis, systemic arterial and pulmonary artery pressures, classification CCS and systolic dysfunction showed statistical significance.

**Conclusions:**

There was no association between cardiac death and valvular AF. Independent predictors of cardiac death were low measures of blood pressure, higher score CCS classification and the presence of systolic ventricular dysfunction.

## Introduction

Atrial fibrillation (AF) affects 2% of the population, its prevalence increases with age, reaching the rate of 15% in those with 80 years, and half of the patients with AF present age equal to or greater than 75 years.^1,2^ Beyond this epidemiological importance, this arrhythmia is associated with worsening of quality of life and tolerance to efforts, thromboembolic phenomena, hospitalization, heart failure (HF), and double the mortality rate.^1,3-6^ AF increases the risk of stroke by 5 times, which also increases with age, with a risk of 1.5% in those between 50 and 59 years of age and 23.5% in the 80-89 age group.^1,3^ Among patients with AF rheumatic valve etiology, the risk is 17 times that of the general population and 5 times in relation to patients with non-valvular AF.^7^ Due to the risk of thromboembolism, oral anticoagulation is indicated for these patients. However, this therapy has complications, with an annual incidence of bleeding of 2.1 per 100 individuals, resulting in a mortality rate between 13 and 33%.^8,9^ Therefore, adequate stratification of the risk of thromboembolism and bleeding is mandatory.

Rheumatic valvulopathy is a disease with a high prevalence in developing countries, yet it is still neglected.^10^ In AF, this etiology presents different percentages, from 2.2% in developed countries to 31.5% in those in development.^11^ There is scarce current data on the treatment and evolution of patients with AF and rheumatic heart disease.^11-13^ Therefore, the objectives of this study are to analyze the clinical profile, thromboembolism and bleeding scores of patients with AF from a single university institution and verify the predictive variables of cardiac mortality.

## Methods

It is an observational, longitudinal and prospective study. The population consisted of 302 consecutive patients with AF, who came from the outpatient clinic and the Cardiology nursing ward and who accepted to participate in the study. Patients were selected over a one-year period. The research project was approved by the Ethics and Research Committee of the institution and all patients signed the Informed Consent Term. Patients underwent clinical evaluation, 12-lead electrocardiogram, transthoracic echocardiography, and clinical pathology exams. The diagnosis of AF was made by electrocardiogram at the time of symptoms or by virtue of irregular heart rhythm. At the time of inclusion in the study, the following scores were calculated for all patients: *American College of Cardiology* (ACC),^7^ HAS-BLED and ATRIA bleeding scores,^14,15^ and severity ratings of symptoms and impact in the quality of life of the *Canadian Cardiovascular Society* (CCS)^16^ and the *European Heart Rhythm Association* (EHRA),^1^ and the Framingham score^17^ for predicting stroke (F1) and for predicting death or stroke (F2). On the other hand, the CHADS_2_, R_2_CHADS_2_ and CHA_2_DS_2_-VAS_C_^1,14,18^ scores were calculated only for non-valvular AF cases. The clinical intercurrences were recorded as thromboembolic events, hemorrhagic events and cardiac death.

For the analysis of the data, the SPSS program (*Statistical Package for Social Science*) version 14.0 was used. The results were expressed in numbers and proportions, for categorical variables, and in measures of central tendency (mean or median) and dispersion (standard deviation) for continuous variables. The Mann-Whitney and chi-square or Fisher tests were used to compare the differences between the continuous and categorical variables, respectively. Survival analysis was performed using the Kaplan-Meier curve, considering the occurrence of cardiac death. Logistic regression analysis was used by the *Stepwise* method, with the dependent variable being the occurrence of cardiac death, considering the variables with p ≤ 0.10 in the univariate analysis. The level of statistical significance adopted was 5%.

## Results

### Population characteristics, thromboembolism and bleeding scores

The casuistry consisted of 302 patients who were followed for 12.8 ± 11.8 months (from 15 days to 66 months), of which 161 (53.3%) were female. The mean age was 58.1 ± 15.1 years, ranging from 18 to 92 years. Clinical data, calculated scores and echocardiographic values are shown in Table 1. They had systolic ventricular dysfunction, defined as the ejection fraction lower than 50%, 112 patients (37.1%). Valvular heart disease was of rheumatic etiology in 96 patients. The valvular dysfunction of rheumatic etiology was moderate/important mitral stenosis in 34 patients, moderate/severe mitral regurgitation in 10, double mitral lesion in 13, and 39 patients had been submitted before to the implantation of a prosthesis in the mitral position, being 10 with mechanical prosthesis . Three patients had mitral valve prolapse with moderate/severe insufficiency. Among patients with non-valvular AF were not included patients with valvular morphological alterations.

**Table 1:** Clinical characteristics and echocardiographic parameters of patients

Variable	Number (proportion and variation)
Femenine gender (%)	161 (53%)
Age (years)	58.1 ± 15.1 (18-92)
BMI (Kg/m^2^)	25.1 ± 5.5 (14.9-55.0)
Paroxysmal AF	87 (28.8%)
Persistent AF	45 (14.9%)
Permanent AF	170 (56.2%)
**Etiology**	
Valvular Heart Disease	99 (32.8%)
Dilated cardiomyopathy	95 (31.5%)
Hypertensive cardiomyopathy	85 (28.1%)
Others (ischemic without ventricular disfunction, congenit, pericarditis constrictive, Brady-Taqui syndrome)	11 (3.6%)
Isolated AF	12 (4.0%)
Previous thromboembolism	62 (20.5%)
HR (bpm)	81 ± 19 (34-180)
SBP (mmHg)	121 ± 22 (60-200)
DBP (mmHg)	75 ± 13 (30-120)
ACC Score	
Low risk	25 (8.6%)
Moderate risk	133 (44.0%)
High risk	143 (47.4%)
CHADS_2_	1.7 ± 1.1 (0-5)
R_2_CHADS_2_	2.5 ± 1.7 (0-7)
CHA_2_DS_2_-VAS_c_	2.9 ± 1.8 (0-8)
F1 (%)	11.8 ± 8.8 (4-54)
F2 (%)	29.7 ± 21.4 (7-95)
CCS	2.6 ± 1.1 (0-4)
EHRA	2.7 ± 0.9 (1-4)
LA (mm)	50.7 ± 10.0 (30-84)
LVDD (mm)	55.5 ± 10.4 (33-86)
LVSD (mm)	40.6 ± 12.9 (17-81)
PSAP (mmHg)	43.6 ± 13.8 (10-101)
LVEF (Teicholz)	51.6 ± 17.3 (12-85)

BMI: body mass index; HR: supine heart rate; bpm: beats per minute; SBP: supine systolic blood pressure; DBP: supine diastolic blood pressure; ACC: American College of Cardiology; Framingham score for prediction of stroke (F1) and prediction of death or stroke (F2); CCS: Canadian Cardiovascular Society; EHRA: European Heart Rhythm Association; AE: anteroposterior diameter of the left atrium; LV: left ventricle; LVDD: LV diastolic diameter; LVSD: LV systolic diameter; PSAP: pulmonary artery systolic pressure; EF: ejection fraction.

Clinical pathology examinations at the time of inclusion of the patients showed the following mean values: creatinine of 1.2 ± 1.1 mg/dL (ranging from 0.3 to 11.4), creatinine clearance by the Cockroft formula And Gault of 72.2 ± 36.4 mL/min (between 4.4 and 233.7), serum sodium of 137.4 ± 4.2 (120.0 to 150.0) mmol/L and serum potassium of 4.2 ± 0.6 (1.3 to 6.3) mmol/L.

At the time of inclusion in the study, 174 patients (57.6%) were using warfarin. The HAS-BLED and ATRIA scores presented the mean values of 1.4 ± 1.1 and 1.2 ± 1.5 (median of 1.0), respectively. In 58 patients (19.2%), the HAS-BLED was ≥ 3 and in 14 patients (4.6%) the ATRIA was high risk.

Eighty patients were on antiarrhythmic medication (4 on sotalol, 11 on propafenone and the rest on amiodarone).

### Clinical follow-up and survival curves

Patients who presented with electrolytic and metabolic disorders were treated according to their disorders. There was no interference of the researchers regarding the approach and therapies adopted by the attending physicians. For heart rate control in those patients with persistent or permanent AF, beta-blockers, or calcium channel antagonists (verapamil or diltiazem) and/or digoxin were used.

During clinical follow-up of 12.8 ± 11.2 (between 15 days and 66 months), 181 (59.9%) patients used warfarin. The International Normalized Ratio (INR) fraction within the therapeutic range (TTR) was calculated to be 45.8 ± 27.6% (between zero and 100%), and 22.9% of the patients evolved with TTR ≥ 60 %. The mean scores of CHADS_2_ and CHA_2_DS_2_-VASC among patients who did not use and those who used warfarin were 1.8 versus 1.6 (p = 0.22) and 3.2 versus 2.6 (p = 0, 12), respectively.

Thirty patients (9.9%) died from cardiac cause and 41 (22.6%) presented some type of hemorrhage due to the use of warfarin. The causes of cardiac death were: HF in 25 patients (83.3%), sudden cardiac death in 3 (10%) and thrombosis in a mechanical valve prosthesis in 2 (6.6%). Only 6 patients (2%) had a new nonfatal thromboembolic event, and 2 were not in regular use of warfarin. The comparison of the studied variables between the patients with and without cardiac death were shown in Table 2. There was no influence of the use of antiarrhythmic and the evolution to cardiac death (16.7% in antiarrhythmic use had cardiac death and 27.5 % in antiarrhythmic use did not present with cardiac death, p = 0.14).

**Table 2:** Comparison of the means and proportions of the variables among the group of patients who attended without and with cardiac death

Variables	Group without CD (n = 272)	Group with CD (n = 30)	Valor p
Age (Years)	58.7 ± 15.1	53.7 ± 13.8	0.14
Femenine gender	146 (53.6%)	15 (50.0%)	0.59
BMI (Kg/m^2^)	25.3 ± 5.3	24.2 ± 6.3	0.13
Permanent AF	146 (53.7%)	24 (80.0%)	0.01
Valvular AF	93 (34.1%)	6 (20.0%)	0.11
HR (bpm)	81.0 ± 19.0	80.3% ± 16.7	0.93
SBP (mmHg)	123.7 ± 20.8	102.0 ± 20.1	< 0.0001
DBP (mmHg)	75.7 ± 13.2	68.1 ± 13.0	0.004
LA (mm)	49.7 ± 9.4	57.9 ± 12.0	0.001
LVDD (mm)	54.7 ± 9.8	64.3 ± 12.3	< 0.0001
LVSD (mm)	39.5 ± 12.2	52.2 ± 14.7	< 0.0001
PASP (mmHg)	42.3 ± 13.3	51.3 ± 12.8	< 0.0001
LVEF (%)	53.2 ± 16.4	37.0 ± 18.4	< 0.0001
Creatinine (mg/dL)	1.2 ± 1.1	1.4 ± 0.6	0.004
Creatinine Clearance (mL/min)	72.8 ± 37.2	57.5 ± 26.5	0.01
Sodium (mmol/L)	137.9 ± 3.9	134.3 ± 4.5	< 0.0001
Potassium (mmol/L)	4.2 ± 0.5	4.1 ± 0.8	0.23
Varfarine use	149 (54.7%)	19 (63.3%)	0.37
TTR	126 (46.3%)	13 (42.3%)	0.66
BP	57 (21.0%)	11 (36.3%)	0.10
CCS	2.5 ± 1.1	3.1 ± 1.0	0.01
EHRA	2.7 ± 0.9	3.2 ± 0.9	0.02
High risk ACC	130 (47.7%)	9 (30.0%)	0.10
CHADS_2_	1.7 ± 1.2	1.5 ± 0.7	0.60
R_2_CHADS_2_	2.4 ± 1.7	3.0 ± 1.1	0.02
CHA_2_DS_2_–VAS_C_	2.9 ± 1.8	2.6 ± 1.4	0.59
F1 (%)	12.0 ± 8.6	8.5 ± 5.3	0.03
F2 (%)	30.1 ± 22.0	26.2 ± 14.1	0.90
HAS-BLED	1.4 ± 1.1	1.8 ± 0.9	0.01
ATRIA	1.2 ± 1.6	1.0 ± 1.1	0.68

CD: cardiac death; BMI: body mass index; HR: supine heart rate; Bpm: beats per minute; SBP: supine systolic blood pressure; DBP: supine diastolic blood pressure; Pts: patients; ACC: score of the American College of Cardiology; Framingham score for prediction of stroke (F1) and prediction of death or stroke (F2); CCS: Canadian Cardiovascular Society; EHRA: European Heart Rhythm Association; LA: anteroposterior diameter of the left atrium; LVDD: LV diastolic diameter; LVSD: LV systolic diameter; PSAP: pulmonary artery systolic pressure; EF: ejection fraction; LV: left ventricle; TTR: fraction of the RNI values (international normalized ratio) within the therapeutic range; BP: bleeding patients.

**Figure 1:**
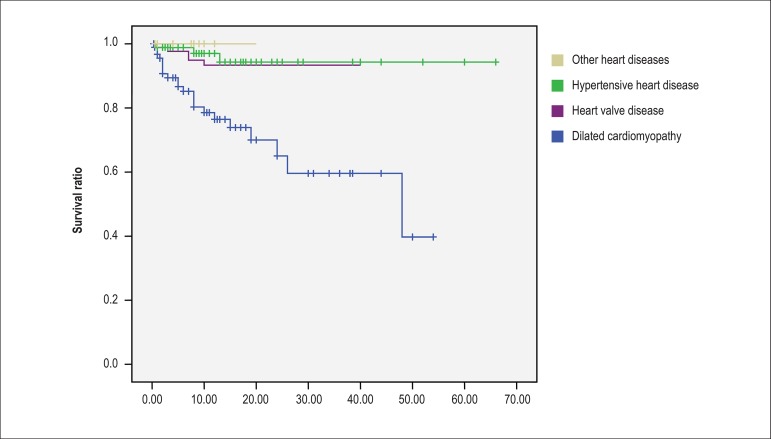
p <0.0001 Kaplan-Meier curve of cardiac death free survival of patients in relation to baseline heart disease.

**Figure 2:**
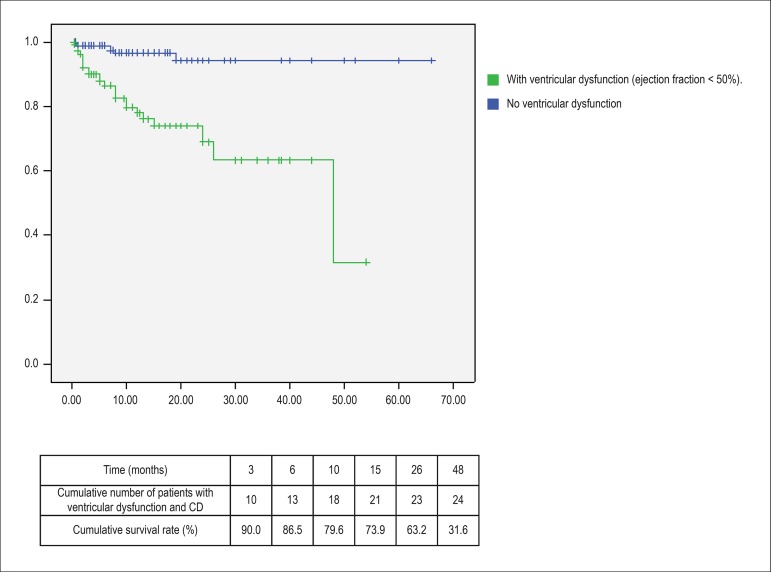
Kaplan-Meier curve and cumulative percentage of cardiac death free survival (CD) of the patients in relation to the presence of systolic ventricular dysfunction.

**Figure 3:**
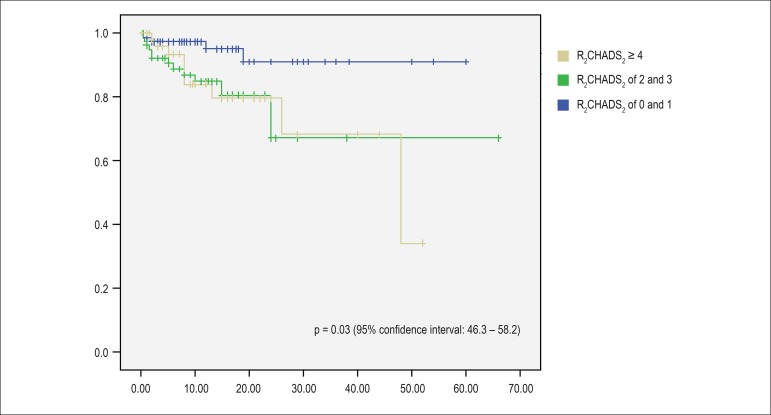
Kaplan-Meier curve of free survival of cardiac death (CD) of patients in relation to the stratification of the score R_2_CHADS_2_.

Among patients who died due to heart failure, 24 (80%) had permanent AF (p = 0.02 by the log rank test (Mantel-Cox), 95% confidence interval between 39.7 and 47.7). Using the Kaplan-Meier curve and considering as a prognostic basis the occurrence of cardiac death, survival curves were constructed in relation to the following variables: baseline heart disease, presence of systolic ventricular dysfunction and stratification of the score R_2_CHADS_2_ (low risk: score of 0 and 1, intermediate risk: 2 and 3, high risk: ≥ 4). Twenty-two patients (73.3%) who progressed to cardiac death had dilated myocardiopathy, 5 rheumatic heart valve disease and 3 hypertensive heart diseases. The Mantel-Cox test was applied to compare the curves. In relation to systolic ventricular dysfunction, the odds ratio was 8.1 (p < 0.0001) (95% confidence interval: 3.2-20.7). The data are plotted in Figures 1, 2 and 3. There was no difference in survival curves regarding the HAS-BLED, EHRA and CCS classification.

### Multivariate analysis

Through the multivariate analysis by *Stepwise* and considering the variables with p ≤ 0.10 in the univariate analysis associated with cardiac death, the variables systemic arterial pressure (systolic and diastolic), pulmonary artery systolic pressure, CCS classification and systolic ventricular dysfunction were statistically significant (Table 3).

**Table 3:** Multivariate analysis for the dependent variable cardiac death

Independent variables	Valor p
SBP (mmHg)	0.001
DBP (mmHg)	0.033
CCS Classification	0.002
PSAP (mmHg)	0.006
Systolic Disfunction LV (EF < 0.50)	0.044

SBP: supine systolic blood pressure; DBP: supine diastolic blood pressure; CCS: Canadian Cardiovascular Society; PSAP: pulmonary artery systolic blood pressure; LV: left ventricle.

## Discussion

The characteristics of the population of the present study were distinct from records already published^19,20^ in relation to age, gender, and mainly regarding the proportion of patients with valvular AF, which was 32.7%. The previous registries were multicentric and performed in developed countries, with only 4.2% of patients with valvar AF, which implied in older age (71.5 and 75 years) and a higher proportion of men (60.1% and 57 %). Due to these factors, the mean of thromboembolism and bleeding scores reported in those registries were also higher, considering the increase in the prevalence of atherosclerosis and blood pressure levels with increasing age. However, the EHRA classification was similar, since 70% of patients in the European registry^19^ presented EHRA II or III, as well as the proportion of patients with heart failure between 21.3% and 36% in the cited registries and 31,4% in the present study.

Underutilization of oral anticoagulant is an aspect already reported in the literature, as well as subtherapeutic treatment, with low rates of TTR,^21-23^ with improved adherence to therapy over time, as demonstrated by the registries studies.^19,20^ Among patients at high risk, with a previous stroke or transient ischemic stroke, about half (ranging from 19% to 81.3%) were not treated with anticoagulants.^22^ In the recent published European registry^19^ and with a previous history of embolism in 15.5% of patients, 78% were in use of some vitamin K antagonist and 6.1% in the use of new oral anticoagulants, with 13.5% of patients with labile INRs. Accordingly, in the American registry,^20^ the mean TTR was 65%, with 17% of patients with INR below the therapeutic range, also showing a greater adherence to the guidelines. In the present study, although about 60% of patients used warfarin at the time of inclusion and during follow-up, one-third of the patients had AF valvular and 20.5% had a history of previous embolism, with TTR lability being observed in 77, 1% of the patients, evidencing an inadequate adherence to the treatment. This underutilization of oral anticoagulation was also verified in a survey conducted in an African country with 25.6% of patients with valvular heart disease and 34.2% with anticoagulant use among eligible patients.^24^

Contemporary data showed an annual mortality rate of 5.8% attributed to AF, reaching up to 8.3%, and that 57.4% of these deaths were of cardiac cause, with 77.3% of them due to HF.^25^ In the present study, there was 9.9% of cardiac mortality, with 83% due to heart failure, of which 80% had permanent AF. In addition, survival curves showed higher cardiac mortality among patients with systolic ventricular dysfunction, with a odds ratio of 8.1. This concomitance of AF and HF, with more than half of the patients with AF having HF and more than one third of HF patients with AF,^26^ translating, a vicious circle adversely influences the prognosis. Systematic review and meta-analysis have confirmed the association between mortality and systolic dysfunction in patients with AF, compared with that of patients with AF, but without systolic dysfunction, during the 2-year period.^27^ In addition, the permanent AF presentation was the most frequent among those with cardiac death in the European multicenter study with a one-year follow-up.^25^

The R_2_CHADS_2_ score was published in 2013^28^ and is calculated by adding 2 more points to CHADS^2^ for patients with non-valvular AF and creatinine clearance < 60 mL/min to better stratify the risk of thromboembolic events. There are no studies on this score and cardiac mortality in patients with AF. In the study in question, the R_2_CHADS_2_ score discriminated patients who presented cardiac death, unlike the other CHADS^2^ and CHA^2^DS^2^-VAS_C_ scores. There is influence of age, gender, ethnicity and weight in the estimation of renal function. The Cockroft & Gault^29^ formula was developed with a population of 249 patients aged 18 to 92 years, which is the same range as the population of the present study. A study of 925 patients with a mean age of 69 years, ranging from 59 to 75.5 years, comparing the three formulas (Cockroft-Gault, MDRD-4 (Modification of Diet in Renal Disease Study), and CKD-EPI (Chronic Kidney Disease Epidemiology Collaboration) demonstrated that the first presented greater accuracy, including comparing groups with EF <40% and ≥40%.^30^ Regarding the R_2_CHADS_2_ score, a recent study^31^ with 524 patients with AF demonstrated its utility in predicting stroke also in patients with impaired renal function, compared with the CHADS^2^ and CHA^2^DS^2^-VAS_C_ scores.

Although several variables were associated with cardiac death, the independent predictors of this evolution were systemic arterial pressure, CCS classification, systolic dysfunction, and pulmonary artery systolic blood pressure. A cohort study^32^ with patients with AF demonstrated that baseline systolic blood pressure < 120 mmHg was associated with cardiovascular mortality in those with systolic ventricular dysfunction during an average follow-up of 41 months, corroborating the results of the present study. In a systematic review with patients with HF, the highest systolic blood pressure was a favorable prognostic marker.^33^

Regarding the CCS classification, which was validated in terms of the quality of life of patients with AF,^34^ its value as an independent variable is due to its graduation, since patients with symptoms of HF secondary to arrhythmia are classified in category 4. On the other side, despite the association between the EHRA score and cardiac death, it was not a predictor of that outcome. This finding was also reported in another cohort of patients with AF, with an association between the score and hospitalization, rather than mortality.^35^

Pulmonary hypertension has been associated with morbidity and mortality, including in those patients with bordering systolic pressure of the pulmonary artery.^36,37^ Its most prevalent cause is left ventricular heart disease, with decreased or preserved ejection fraction, and mitral valvopathy. Therefore, this finding in the present study demonstrated what is already reported in the literature.

### Limitations of the study

The main limitations of this study are the size of the population and the fact that it is unicentric, which does not reflect the disparities in the approach of these patients between institutions and regions. In addition, the influence of interventions such as arrhythmia reversal or ablation in patients' evolution was not investigated.

## Conclusions

In the population with AF and high prevalence of rheumatic valve disease, there was an underutilization of oral anticoagulant, in spite of lower bleeding scores and thromboembolism in relation to those reported in the literature. Survival was lower in those with permanent AF, with dilated cardiomyopathy, and with high-risk R_2_CHADS_2_. The independent predictors of cardiac death were low measures of systemic arterial pressure, higher CCS scores, presence of systolic ventricular dysfunction, and pulmonary hypertension.
